# Work-style Reform: Considering Not Only Residents but Also Senior Staff

**DOI:** 10.31662/jmaj.2024-0082

**Published:** 2024-06-28

**Authors:** Hironori Takahashi, Shigeki Matsubara

**Affiliations:** 1 Department of Obstetrics and Gynecology, Jichi Medical University, Tochigi, Japan

**Keywords:** academic career, resident, supervisor, work-style reform

## Dear Editors:

We thank Saeki and colleagues ^(1)^ for their comments. We previously wrote that 1) demanding publication during residency for a Specialist Certificate may prompt residents to submit papers to non-PubMed journals, precluding data from worldwide readers’ recognition ^(2)^, and 2) when attending residents do not write case reports, senior doctors should take their place ^(3)^, with increasing senior doctors’ working hours and thus their burden. Saeki et al. emphasized that the work-style reform introduction (*Hatarakikata-Kaikaku*) may have some negative impacts on research activity. Importantly, they focused on senior doctors’ situations: the reform may increase senior doctors’ burden. They proposed the following: i) task shift to the nondoctor staff, ii) evaluating academic achievement, and iii) giving incentives to senior doctors for mentoring residents’ research. We agree with their views.

Senior doctors are struggling. Let us focus on paper writing and consider the present situation as an example. Creating this 400-word letter, from reading Saeki’s Letter to submission completion, required approximately 25 hours for the first author. Writing left to a resident alone may take four times this at 100 hours, exceeding the upper limit of monthly work (*Zangyo*). In this extreme scenario, the resident must perform clinical work from 9 a.m. to 5 p.m. and then dash to write a paper, using the permitted 100 hours. It is important to consider that a case report typically consists of 1000-1500 words, which may require even more time to complete.

To avoid the worst-case scenario in which the resident’s entire 100 hours are allocated to paper writing, senior doctors are obligated to help or alternate with residents in paper writing, at least to some extent. Consider that senior doctors do not operate under the 100-hour regulation but rather under the discretionary work system (*Sairyoroudousei*), where wages are accrued based on predetermined working hours, and actual working hours are not strictly regulated like those of residents.

We, seasoned doctors, have tended, and still tend, to care less about working extra hours. We have grown in an environment of “daytime clinician, night-time researcher.” This may be one of the background reasons why some residents work themselves to exhaustion (*Karoshi*). Although unintentionally, older doctors may have contributed to this situation. Now, we must discard the nostalgia for the “the harder, the better” era. Instead, senior doctors should be appropriately treated and incentivized regarding working hours. Nobody wishes for senior doctors to suffer from *Karoshi*.

## Article Information

### Conflicts of Interest

None

### Author Contributions

HT and SM identified the significance and wrote the manuscript.

### Approval by Institutional Review Board (IRB)

Not needed.

### Informed Consent

Not applicable.

### Patient Anonymity

Not applicable.
